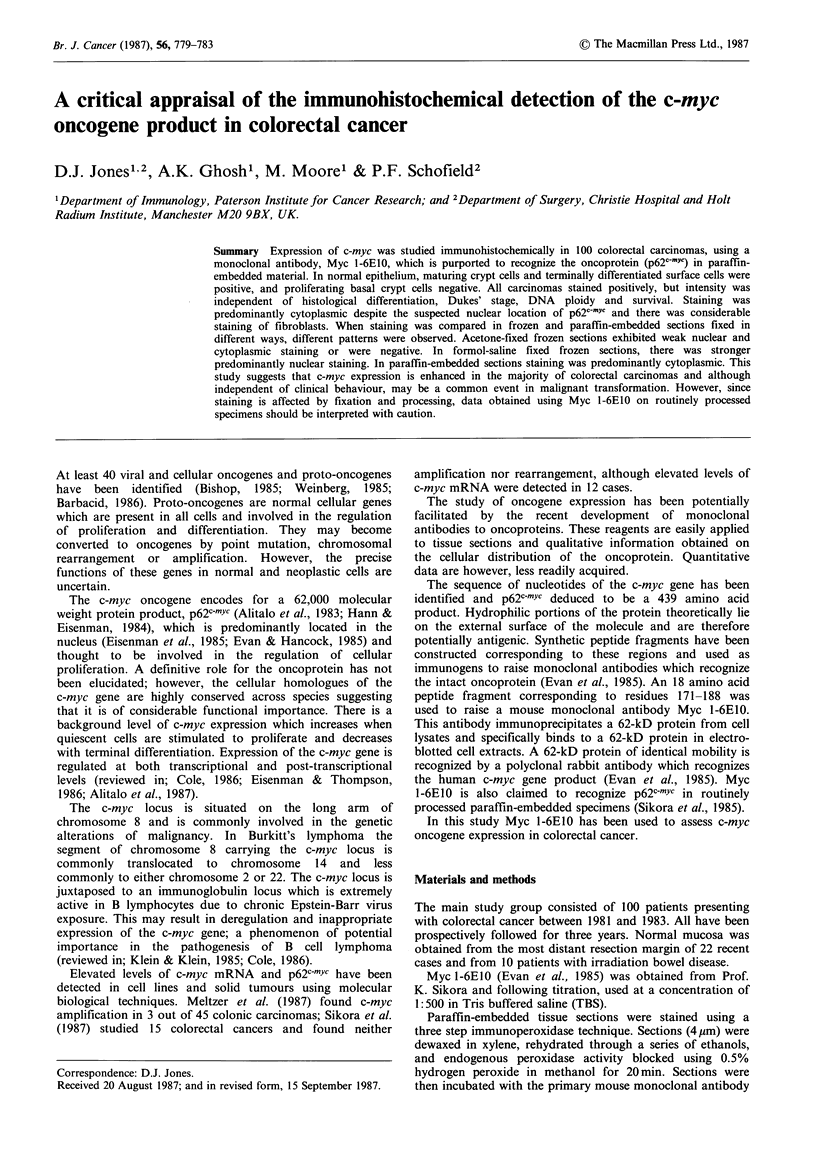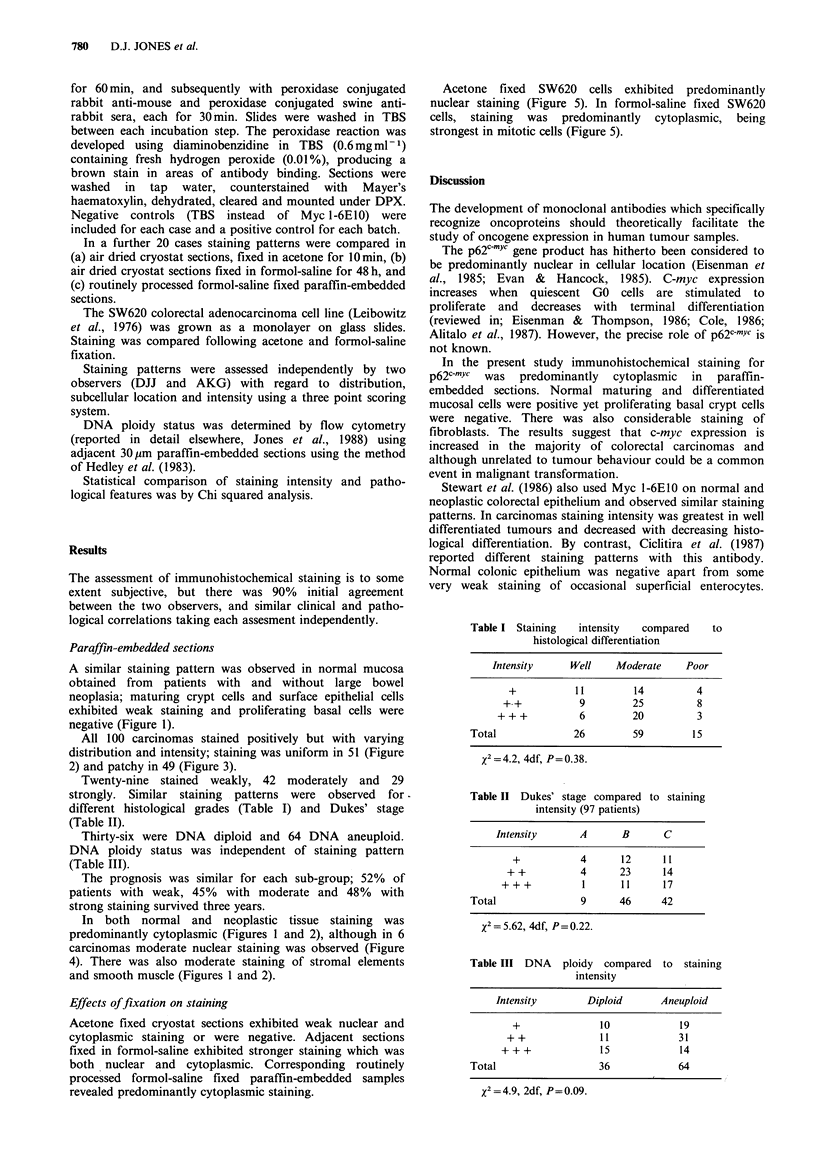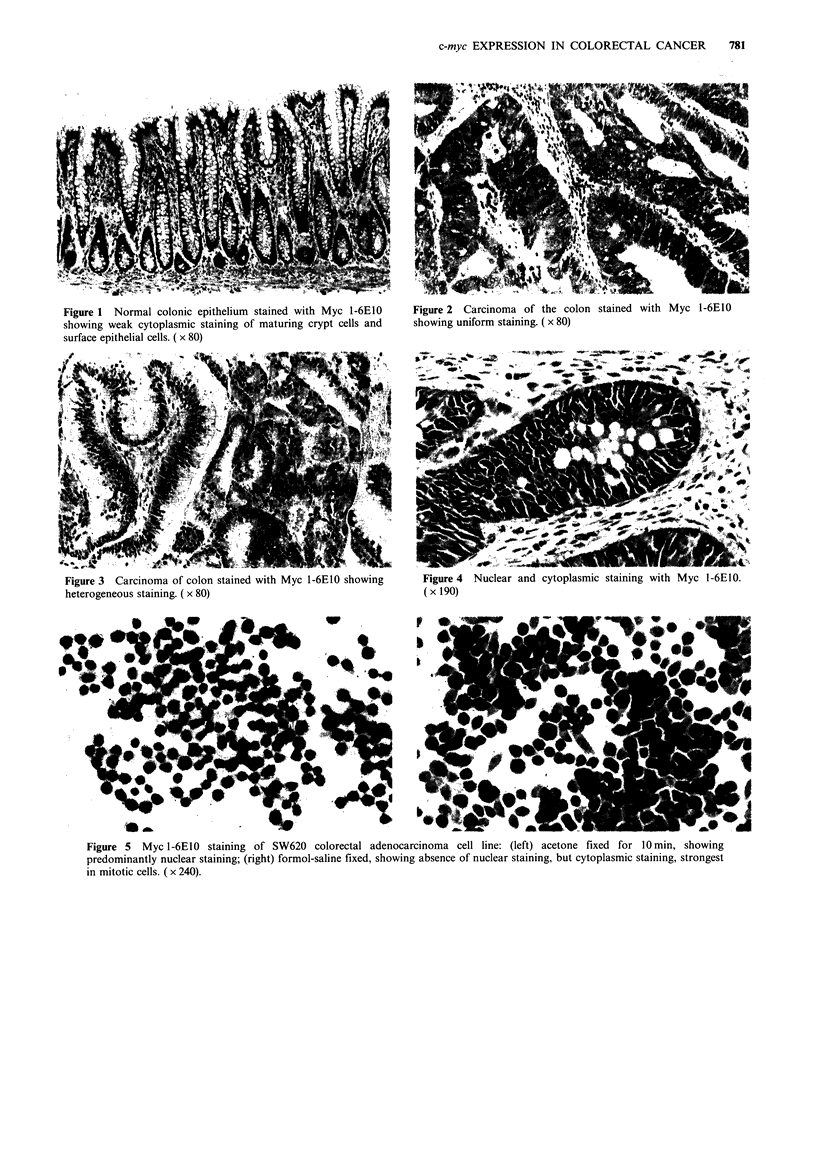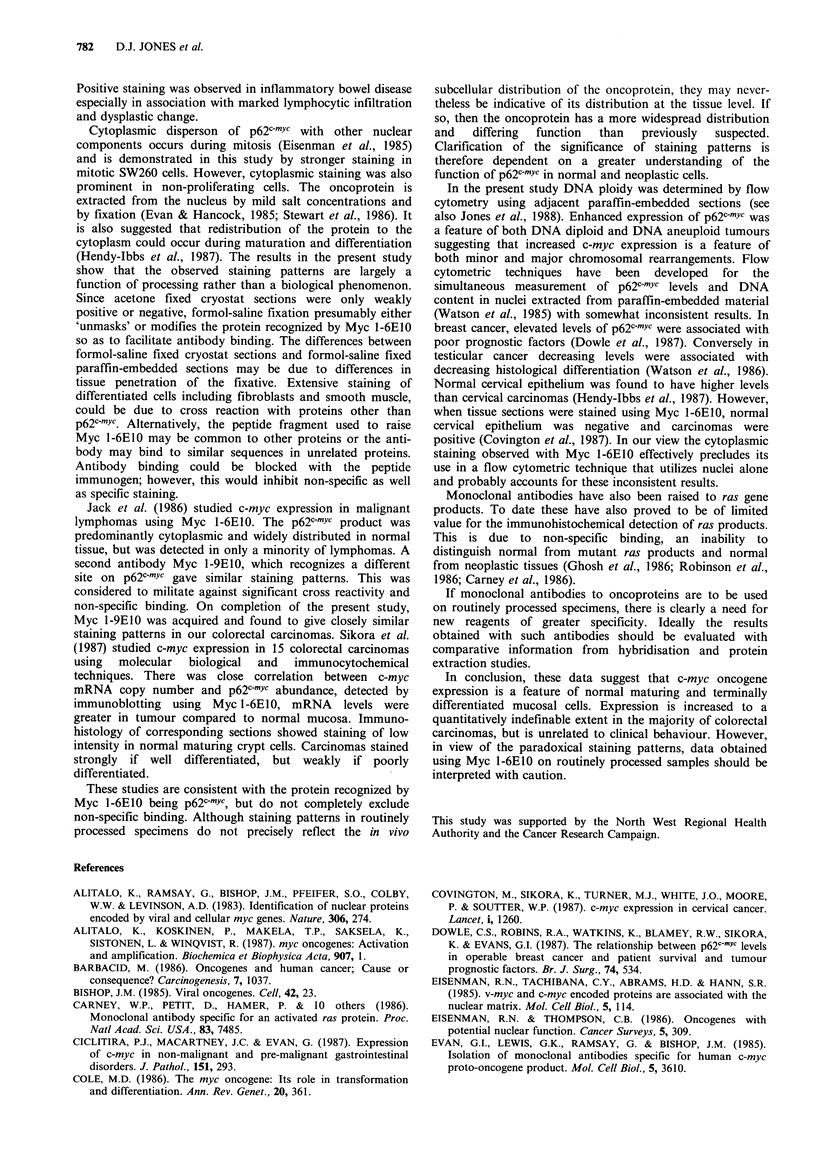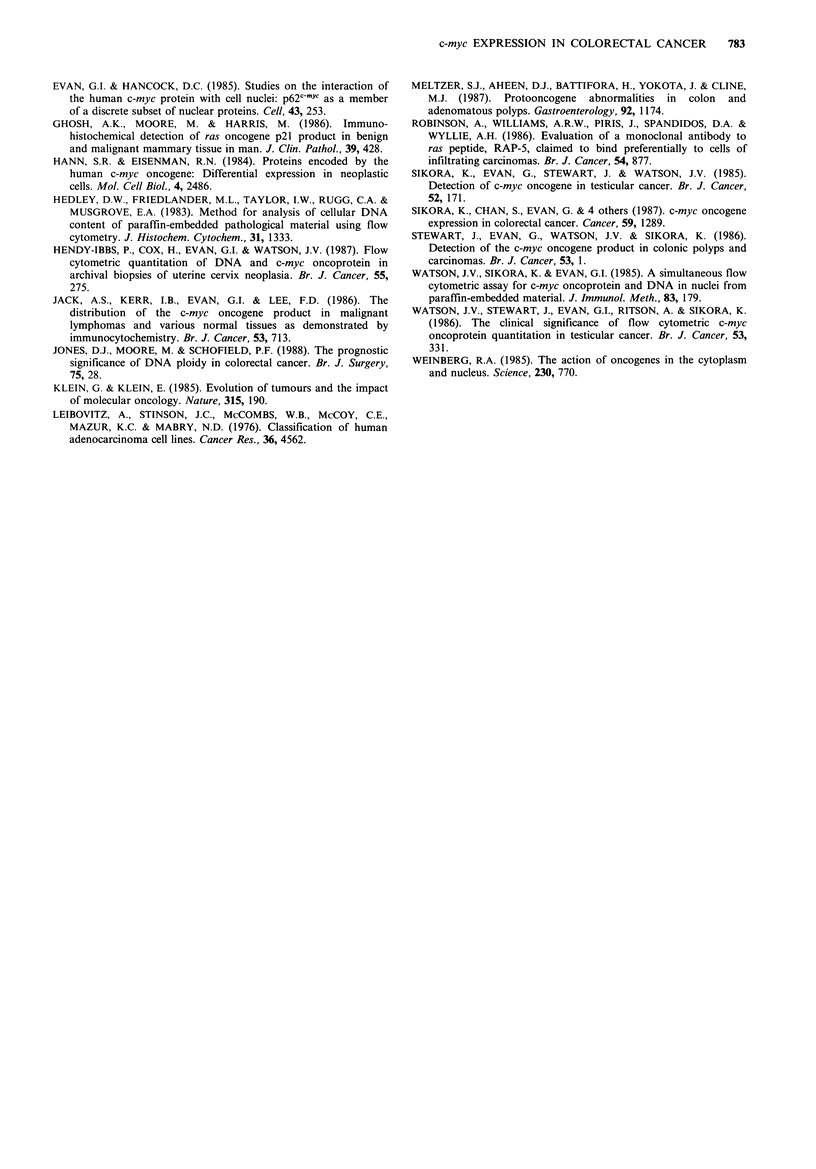# A critical appraisal of the immunohistochemical detection of the c-myc oncogene product in colorectal cancer.

**DOI:** 10.1038/bjc.1987.287

**Published:** 1987-12

**Authors:** D. J. Jones, A. K. Ghosh, M. Moore, P. F. Schofield

**Affiliations:** Department of Immunology, Paterson Institute for Cancer Research, Manchester, UK.

## Abstract

**Images:**


*Br. J. Cancer* (1987), **56**, 779–783 © The Macmillan Press Ltd., 1987

### A critical appraisal of the immunohistochemical detection of the c-*myc* oncogene product in colorectal cancer

D.J. Jones[1,2], A.K. Ghosh[1], M. Moore[1] & P.F. Schofield[2]

[1]*Department of Immunology, Paterson Institute for Cancer Research; and* [2]*Department of Surgery, Christie Hospital and Holt Radium Institute, Manchester M20 9BX, UK.*

**Summary** Expression of c-*myc* was studied immunohistochemically in 100 colorectal carcinomas, using a monoclonal antibody, Myc 1-6E10, which is purported to recognize the oncoprotein (p62$^{c\text{-}myc}$) in paraffin-embedded material. In normal epithelium, maturing crypt cells and terminally differentiated surface cells were positive, and proliferating basal crypt cells negative. All carcinomas stained positively, but intensity was independent of histological differentiation, Dukes' stage, DNA ploidy and survival. Staining was predominantly cytoplasmic despite the suspected nuclear location of p62$^{c\text{-}myc}$ and there was considerable staining of fibroblasts. When staining was compared in frozen and paraffin-embedded sections fixed in different ways, different patterns were observed. Acetone-fixed frozen sections exhibited weak nuclear and cytoplasmic staining or were negative. In formol-saline fixed frozen sections, there was stronger predominantly nuclear staining. In paraffin-embedded sections staining was predominantly cytoplasmic. This study suggests that c-*myc* expression is enhanced in the majority of colorectal carcinomas and although independent of clinical behaviour, may be a common event in malignant transformation. However, since staining is affected by fixation and processing, data obtained using Myc 1-6E10 on routinely processed specimens should be interpreted with caution.

At least 40 viral and cellular oncogenes and proto-oncogenes have been identified (Bishop, 1985; Weinberg, 1985; Barbacid, 1986). Proto-oncogenes are normal cellular genes which are present in all cells and involved in the regulation of proliferation and differentiation. They may become converted to oncogenes by point mutation, chromosomal rearrangement or amplification. However, the precise functions of these genes in normal and neoplastic cells are uncertain.

The c-*myc* oncogene encodes for a 62,000 molecular weight protein product, p62$^{c\text{-}myc}$ (Alitalo *et al.*, 1983; Hann & Eisenman, 1984), which is predominantly located in the nucleus (Eisenman *et al.*, 1985; Evan & Hancock, 1985) and thought to be involved in the regulation of cellular proliferation. A definitive role for the oncoprotein has not been elucidated; however, the cellular homologues of the c-*myc* gene are highly conserved across species suggesting that it is of considerable functional importance. There is a background level of c-*myc* expression which increases when quiescent cells are stimulated to proliferate and decreases with terminal differentiation. Expression of the c-*myc* gene is regulated at both transcriptional and post-transcriptional levels (reviewed in; Cole, 1986; Eisenman & Thompson, 1986; Alitalo *et al.*, 1987).

The c-*myc* locus is situated on the long arm of chromosome 8 and is commonly involved in the genetic alterations of malignancy. In Burkitt's lymphoma the segment of chromosome 8 carrying the c-*myc* locus is commonly translocated to chromosome 14 and less commonly to either chromosome 2 or 22. The c-*myc* locus is juxtaposed to an immunoglobulin locus which is extremely active in B lymphocytes due to chronic Epstein-Barr virus exposure. This may result in deregulation and inappropriate expression of the c-*myc* gene; a phenomenon of potential importance in the pathogenesis of B cell lymphoma (reviewed in; Klein & Klein, 1985; Cole, 1986).

Elevated levels of c-*myc* mRNA and p62$^{c\text{-}myc}$ have been detected in cell lines and solid tumours using molecular biological techniques. Meltzer *et al.* (1987) found c-*myc* amplification in 3 out of 45 colonic carcinomas; Sikora *et al.* (1987) studied 15 colorectal cancers and found neither amplification nor rearrangement, although elevated levels of c-*myc* mRNA were detected in 12 cases.

The study of oncogene expression has been potentially facilitated by the recent development of monoclonal antibodies to oncoproteins. These reagents are easily applied to tissue sections and qualitative information obtained on the cellular distribution of the oncoprotein. Quantitative data are however, less readily acquired.

The sequence of nucleotides of the c-*myc* gene has been identified and p62$^{c\text{-}myc}$ deduced to be a 439 amino acid product. Hydrophilic portions of the protein theoretically lie on the external surface of the molecule and are therefore potentially antigenic. Synthetic peptide fragments have been constructed corresponding to these regions and used as immunogens to raise monoclonal antibodies which recognize the intact oncoprotein (Evan *et al.*, 1985). An 18 amino acid peptide fragment corresponding to residues 171–188 was used to raise a mouse monoclonal antibody Myc 1-6E10. This antibody immunoprecipitates a 62-kD protein from cell lysates and specifically binds to a 62-kD protein in electro-blotted cell extracts. A 62-kD protein of identical mobility is recognized by a polyclonal rabbit antibody which recognizes the human c-*myc* gene product (Evan *et al.*, 1985). Myc 1-6E10 is also claimed to recognize p62$^{c\text{-}myc}$ in routinely processed paraffin-embedded specimens (Sikora *et al.*, 1985).

In this study Myc 1-6E10 has been used to assess c-*myc* oncogene expression in colorectal cancer.

### Materials and methods

The main study group consisted of 100 patients presenting with colorectal cancer between 1981 and 1983. All have been prospectively followed for three years. Normal mucosa was obtained from the most distant resection margin of 22 recent cases and from 10 patients with irradiation bowel disease.

Myc 1-6E10 (Evan *et al.*, 1985) was obtained from Prof. K. Sikora and following titration, used at a concentration of 1:500 in Tris buffered saline (TBS).

Paraffin-embedded tissue sections were stained using a three step immunoperoxidase technique. Sections (4 $\mu$m) were dewaxed in xylene, rehydrated through a series of ethanols, and endogenous peroxidase activity blocked using 0.5% hydrogen peroxide in methanol for 20 min. Sections were then incubated with the primary mouse monoclonal antibody

Correspondence: D.J. Jones.
Received 20 August 1987; and in revised form, 15 September 1987.

for 60 min, and subsequently with peroxidase conjugated rabbit anti-mouse and peroxidase conjugated swine anti-rabbit sera, each for 30 min. Slides were washed in TBS between each incubation step. The peroxidase reaction was developed using diaminobenzidine in TBS ($0.6\,mg\,ml^{-1}$) containing fresh hydrogen peroxide (0.01%), producing a brown stain in areas of antibody binding. Sections were washed in tap water, counterstained with Mayer's haematoxylin, dehydrated, cleared and mounted under DPX. Negative controls (TBS instead of Myc 1-6E10) were included for each case and a positive control for each batch.

In a further 20 cases staining patterns were compared in (a) air dried cryostat sections, fixed in acetone for 10 min, (b) air dried cryostat sections fixed in formol-saline for 48 h, and (c) routinely processed formol-saline fixed paraffin-embedded sections.

The SW620 colorectal adenocarcinoma cell line (Leibowitz *et al.*, 1976) was grown as a monolayer on glass slides. Staining was compared following acetone and formol-saline fixation.

Staining patterns were assessed independently by two observers (DJJ and AKG) with regard to distribution, subcellular location and intensity using a three point scoring system.

DNA ploidy status was determined by flow cytometry (reported in detail elsewhere, Jones *et al.*, 1988) using adjacent $30\,\mu m$ paraffin-embedded sections using the method of Hedley *et al.* (1983).

Statistical comparison of staining intensity and pathological features was by Chi squared analysis.

### Results

The assessment of immunohistochemical staining is to some extent subjective, but there was 90% initial agreement between the two observers, and similar clinical and pathological correlations taking each assesment independently.

### *Paraffin-embedded sections*

A similar staining pattern was observed in normal mucosa obtained from patients with and without large bowel neoplasia; maturing crypt cells and surface epithelial cells exhibited weak staining and proliferating basal cells were negative (Figure 1).

All 100 carcinomas stained positively but with varying distribution and intensity; staining was uniform in 51 (Figure 2) and patchy in 49 (Figure 3).

Twenty-nine stained weakly, 42 moderately and 29 strongly. Similar staining patterns were observed for different histological grades (Table I) and Dukes' stage (Table II).

Thirty-six were DNA diploid and 64 DNA aneuploid. DNA ploidy status was independent of staining pattern (Table III).

The prognosis was similar for each sub-group; 52% of patients with weak, 45% with moderate and 48% with strong staining survived three years.

In both normal and neoplastic tissue staining was predominantly cytoplasmic (Figures 1 and 2), although in 6 carcinomas moderate nuclear staining was observed (Figure 4). There was also moderate staining of stromal elements and smooth muscle (Figures 1 and 2).

### *Effects of fixation on staining*

Acetone fixed cryostat sections exhibited weak nuclear and cytoplasmic staining or were negative. Adjacent sections fixed in formol-saline exhibited stronger staining which was both nuclear and cytoplasmic. Corresponding routinely processed formol-saline fixed paraffin-embedded samples revealed predominantly cytoplasmic staining.

Acetone fixed SW620 cells exhibited predominantly nuclear staining (Figure 5). In formol-saline fixed SW620 cells, staining was predominantly cytoplasmic, being strongest in mitotic cells (Figure 5).

### Discussion

The development of monoclonal antibodies which specifically recognize oncoproteins should theoretically facilitate the study of oncogene expression in human tumour samples.

The $p62^{c\text{-}myc}$ gene product has hitherto been considered to be predominantly nuclear in cellular location (Eisenman *et al.*, 1985; Evan & Hancock, 1985). *C-myc* expression increases when quiescent G0 cells are stimulated to proliferate and decreases with terminal differentiation (reviewed in; Eisenman & Thompson, 1986; Cole, 1986; Alitalo *et al.*, 1987). However, the precise role of $p62^{c\text{-}myc}$ is not known.

In the present study immunohistochemical staining for $p62^{c\text{-}myc}$ was predominantly cytoplasmic in paraffin-embedded sections. Normal maturing and differentiated mucosal cells were positive yet proliferating basal crypt cells were negative. There was also considerable staining of fibroblasts. The results suggest that c-*myc* expression is increased in the majority of colorectal carcinomas and although unrelated to tumour behaviour could be a common event in malignant transformation.

Stewart *et al.* (1986) also used Myc 1-6E10 on normal and neoplastic colorectal epithelium and observed similar staining patterns. In carcinomas staining intensity was greatest in well differentiated tumours and decreased with decreasing histological differentiation. By contrast, Ciclitira *et al.* (1987) reported different staining patterns with this antibody. Normal colonic epithelium was negative apart from some very weak staining of occasional superficial enterocytes.

**Table I** Staining intensity compared to histological differentiation

| *Intensity* | *Well* | *Moderate* | *Poor* |
|---|---|---|---|
| + | 11 | 14 | 4 |
| ++ | 9 | 25 | 8 |
| +++ | 6 | 20 | 3 |
| Total | 26 | 59 | 15 |

$\chi^2 = 4.2$, 4df, $P = 0.38$.

**Table II** Dukes' stage compared to staining intensity (97 patients)

| *Intensity* | *A* | *B* | *C* |
|---|---|---|---|
| + | 4 | 12 | 11 |
| ++ | 4 | 23 | 14 |
| +++ | 1 | 11 | 17 |
| Total | 9 | 46 | 42 |

$\chi^2 = 5.62$, 4df, $P = 0.22$.

**Table III** DNA ploidy compared to staining intensity

| *Intensity* | *Diploid* | *Aneuploid* |
|---|---|---|
| + | 10 | 19 |
| ++ | 11 | 31 |
| +++ | 15 | 14 |
| Total | 36 | 64 |

$\chi^2 = 4.9$, 2df, $P = 0.09$.

**Figure 1** Normal colonic epithelium stained with Myc 1-6E10 showing weak cytoplasmic staining of maturing crypt cells and surface epithelial cells. ( × 80)

**Figure 2** Carcinoma of the colon stained with Myc 1-6E10 showing uniform staining. ( × 80)

**Figure 3** Carcinoma of colon stained with Myc 1-6E10 showing heterogeneous staining. ( × 80)

**Figure 4** Nuclear and cytoplasmic staining with Myc 1-6E10. ( × 190)

**Figure 5** Myc 1-6E10 staining of SW620 colorectal adenocarcinoma cell line: (left) acetone fixed for 10 min, showing predominantly nuclear staining; (right) formol-saline fixed, showing absence of nuclear staining, but cytoplasmic staining, strongest in mitotic cells. ( × 240).

Positive staining was observed in inflammatory bowel disease especially in association with marked lymphocytic infiltration and dysplastic change.

Cytoplasmic disperson of p62$^{c-myc}$ with other nuclear components occurs during mitosis (Eisenman *et al.*, 1985) and is demonstrated in this study by stronger staining in mitotic SW260 cells. However, cytoplasmic staining was also prominent in non-proliferating cells. The oncoprotein is extracted from the nucleus by mild salt concentrations and by fixation (Evan & Hancock, 1985; Stewart *et al.*, 1986). It is also suggested that redistribution of the protein to the cytoplasm could occur during maturation and differentiation (Hendy-Ibbs *et al.*, 1987). The results in the present study show that the observed staining patterns are largely a function of processing rather than a biological phenomenon. Since acetone fixed cryostat sections were only weakly positive or negative, formol-saline fixation presumably either 'unmasks' or modifies the protein recognized by Myc 1-6E10 so as to facilitate antibody binding. The differences between formol-saline fixed cryostat sections and formol-saline fixed paraffin-embedded sections may be due to differences in tissue penetration of the fixative. Extensive staining of differentiated cells including fibroblasts and smooth muscle, could be due to cross reaction with proteins other than p62$^{c-myc}$. Alternatively, the peptide fragment used to raise Myc 1-6E10 may be common to other proteins or the antibody may bind to similar sequences in unrelated proteins. Antibody binding could be blocked with the peptide immunogen; however, this would inhibit non-specific as well as specific staining.

Jack *et al.* (1986) studied c-*myc* expression in malignant lymphomas using Myc 1-6E10. The p62$^{c-myc}$ product was predominantly cytoplasmic and widely distributed in normal tissue, but was detected in only a minority of lymphomas. A second antibody Myc 1-9E10, which recognizes a different site on p62$^{c-myc}$ gave similar staining patterns. This was considered to militate against significant cross reactivity and non-specific binding. On completion of the present study, Myc 1-9E10 was acquired and found to give closely similar staining patterns in our colorectal carcinomas. Sikora *et al.* (1987) studied c-*myc* expression in 15 colorectal carcinomas using molecular biological and immunocytochemical techniques. There was close correlation between c-*myc* mRNA copy number and p62$^{c-myc}$ abundance, detected by immunoblotting using Myc 1-6E10, mRNA levels were greater in tumour compared to normal mucosa. Immunohistology of corresponding sections showed staining of low intensity in normal maturing crypt cells. Carcinomas stained strongly if well differentiated, but weakly if poorly differentiated.

These studies are consistent with the protein recognized by Myc 1-6E10 being p62$^{c-myc}$, but do not completely exclude non-specific binding. Although staining patterns in routinely processed specimens do not precisely reflect the *in vivo* subcellular distribution of the oncoprotein, they may nevertheless be indicative of its distribution at the tissue level. If so, then the oncoprotein has a more widespread distribution and differing function than previously suspected. Clarification of the significance of staining patterns is therefore dependent on a greater understanding of the function of p62$^{c-myc}$ in normal and neoplastic cells.

In the present study DNA ploidy was determined by flow cytometry using adjacent paraffin-embedded sections (see also Jones *et al.*, 1988). Enhanced expression of p62$^{c-myc}$ was a feature of both DNA diploid and DNA aneuploid tumours suggesting that increased c-*myc* expression is a feature of both minor and major chromosomal rearrangements. Flow cytometric techniques have been developed for the simultaneous measurement of p62$^{c-myc}$ levels and DNA content in nuclei extracted from paraffin-embedded material (Watson *et al.*, 1985) with somewhat inconsistent results. In breast cancer, elevated levels of p62$^{c-myc}$ were associated with poor prognostic factors (Dowle *et al.*, 1987). Conversely in testicular cancer decreasing levels were associated with decreasing histological differentiation (Watson *et al.*, 1986). Normal cervical epithelium was found to have higher levels than cervical carcinomas (Hendy-Ibbs *et al.*, 1987). However, when tissue sections were stained using Myc 1-6E10, normal cervical epithelium was negative and carcinomas were positive (Covington *et al.*, 1987). In our view the cytoplasmic staining observed with Myc 1-6E10 effectively precludes its use in a flow cytometric technique that utilizes nuclei alone and probably accounts for these inconsistent results.

Monoclonal antibodies have also been raised to *ras* gene products. To date these have also proved to be of limited value for the immunohistochemical detection of *ras* products. This is due to non-specific binding, an inability to distinguish normal from mutant *ras* products and normal from neoplastic tissues (Ghosh *et al.*, 1986; Robinson *et al.*, 1986; Carney *et al.*, 1986).

If monoclonal antibodies to oncoproteins are to be used on routinely processed specimens, there is clearly a need for new reagents of greater specificity. Ideally the results obtained with such antibodies should be evaluated with comparative information from hybridisation and protein extraction studies.

In conclusion, these data suggest that c-*myc* oncogene expression is a feature of normal maturing and terminally differentiated mucosal cells. Expression is increased to a quantitatively indefinable extent in the majority of colorectal carcinomas, but is unrelated to clinical behaviour. However, in view of the paradoxical staining patterns, data obtained using Myc 1-6E10 on routinely processed samples should be interpreted with caution.

This study was supported by the North West Regional Health Authority and the Cancer Research Campaign.

### References

ALITALO, K., RAMSAY, G., BISHOP, J.M., PFEIFER, S.O., COLBY, W.W. & LEVINSON, A.D. (1983). Identification of nuclear proteins encoded by viral and cellular *myc* genes. *Nature*, **306**, 274.

ALITALO, K., KOSKINEN, P., MAKELA, T.P., SAKSELA, K., SISTONEN, L. & WINQVIST, R. (1987). *myc* oncogenes: Activation and amplification. *Biochemica et Biophysica Acta*, **907**, 1.

BARBACID, M. (1986). Oncogenes and human cancer; Cause or consequence? *Carcinogenesis*, **7**, 1037.

BISHOP, J.M. (1985). Viral oncogenes. *Cell*, **42**, 23.

CARNEY, W.P., PETIT, D., HAMER, P. & 10 others (1986). Monoclonal antibody specific for an activated *ras* protein. *Proc. Natl Acad. Sci. USA.*, **83**, 7485.

CICLITIRA, P.J., MACARTNEY, J.C. & EVAN, G. (1987). Expression of c-*myc* in non-malignant and pre-malignant gastrointestinal disorders. *J. Pathol.*, **151**, 293.

COLE, M.D. (1986). The *myc* oncogene: Its role in transformation and differentiation. *Ann. Rev. Genet.*, **20**, 361.

COVINGTON, M., SIKORA, K., TURNER, M.J., WHITE, J.O., MOORE, P. & SOUTTER, W.P. (1987). c-*myc* expression in cervical cancer. *Lancet*, **i**, 1260.

DOWLE, C.S., ROBINS, R.A., WATKINS, K., BLAMEY, R.W., SIKORA, K. & EVANS, G.I. (1987). The relationship between p62$^{c-myc}$ levels in operable breast cancer and patient survival and tumour prognostic factors. *Br. J. Surg.*, **74**, 534.

EISENMAN, R.N., TACHIBANA, C.Y., ABRAMS, H.D. & HANN, S.R. (1985). v-*myc* and c-*myc* encoded proteins are associated with the nuclear matrix. *Mol. Cell Biol.*, **5**, 114.

EISENMAN, R.N. & THOMPSON, C.B. (1986). Oncogenes with potential nuclear function. *Cancer Surveys*, **5**, 309.

EVAN, G.I., LEWIS, G.K., RAMSAY, G. & BISHOP, J.M. (1985). Isolation of monoclonal antibodies specific for human c-*myc* proto-oncogene product. *Mol. Cell Biol.*, **5**, 3610.

EVAN, G.I. & HANCOCK, D.C. (1985). Studies on the interaction of the human c-*myc* protein with cell nuclei: p62$^{c\text{-}myc}$ as a member of a discrete subset of nuclear proteins. *Cell*, **43**, 253.

GHOSH, A.K., MOORE, M. & HARRIS, M. (1986). Immunohistochemical detection of *ras* oncogene p21 product in benign and malignant mammary tissue in man. *J. Clin. Pathol.*, **39**, 428.

HANN, S.R. & EISENMAN, R.N. (1984). Proteins encoded by the human c-*myc* oncogene: Differential expression in neoplastic cells. *Mol. Cell Biol.*, **4**, 2486.

HEDLEY, D.W., FRIEDLANDER, M.L., TAYLOR, I.W., RUGG, C.A. & MUSGROVE, E.A. (1983). Method for analysis of cellular DNA content of paraffin-embedded pathological material using flow cytometry. *J. Histochem. Cytochem.*, **31**, 1333.

HENDY-IBBS, P., COX, H., EVAN, G.I. & WATSON, J.V. (1987). Flow cytometric quantitation of DNA and c-*myc* oncoprotein in archival biopsies of uterine cervix neoplasia. *Br. J. Cancer*, **55**, 275.

JACK, A.S., KERR, I.B., EVAN, G.I. & LEE, F.D. (1986). The distribution of the c-*myc* oncogene product in malignant lymphomas and various normal tissues as demonstrated by immunocytochemistry. *Br. J. Cancer*, **53**, 713.

JONES, D.J., MOORE, M. & SCHOFIELD, P.F. (1988). The prognostic significance of DNA ploidy in colorectal cancer. *Br. J. Surgery*, **75**, 28.

KLEIN, G. & KLEIN, E. (1985). Evolution of tumours and the impact of molecular oncology. *Nature*, **315**, 190.

LEIBOVITZ, A., STINSON, J.C., McCOMBS, W.B., McCOY, C.E., MAZUR, K.C. & MABRY, N.D. (1976). Classification of human adenocarcinoma cell lines. *Cancer Res.*, **36**, 4562.

MELTZER, S.J., AHEEN, D.J., BATTIFORA, H., YOKOTA, J. & CLINE, M.J. (1987). Protooncogene abnormalities in colon and adenomatous polyps. *Gastroenterology*, **92**, 1174.

ROBINSON, A., WILLIAMS, A.R.W., PIRIS, J., SPANDIDOS, D.A. & WYLLIE, A.H. (1986). Evaluation of a monoclonal antibody to *ras* peptide, RAP-5, claimed to bind preferentially to cells of infiltrating carcinomas. *Br. J. Cancer*, **54**, 877.

SIKORA, K., EVAN, G., STEWART, J. & WATSON, J.V. (1985). Detection of c-*myc* oncogene in testicular cancer. *Br. J. Cancer*, **52**, 171.

SIKORA, K., CHAN, S., EVAN, G. & 4 others (1987). c-*myc* oncogene expression in colorectal cancer. *Cancer*, **59**, 1289.

STEWART, J., EVAN, G., WATSON, J.V. & SIKORA, K. (1986). Detection of the c-*myc* oncogene product in colonic polyps and carcinomas. *Br. J. Cancer*, **53**, 1.

WATSON, J.V., SIKORA, K. & EVAN, G.I. (1985). A simultaneous flow cytometric assay for c-*myc* oncoprotein and DNA in nuclei from paraffin-embedded material. *J. Immunol. Meth.*, **83**, 179.

WATSON, J.V., STEWART, J., EVAN, G.I., RITSON, A. & SIKORA, K. (1986). The clinical significance of flow cytometric c-*myc* oncoprotein quantitation in testicular cancer. *Br. J. Cancer*, **53**, 331.

WEINBERG, R.A. (1985). The action of oncogenes in the cytoplasm and nucleus. *Science*, **230**, 770.